# New Link in the Food Chain? Marine Plastic Pollution and Seafood Safety

**DOI:** 10.1289/ehp.123-A34

**Published:** 2015-02-01

**Authors:** Nate Seltenrich

**Affiliations:** Nate Seltenrich covers science and the environment from Petaluma, CA. His work has appeared in *High Country News*, *Sierra*, *Yale Environment 360*, *Earth Island Journal*, and other regional and national publications.

In recent years plastic pollution in the ocean has become a significant environmental concern for governments, scientists, nongovernmental organizations, and members of the public worldwide. A December 2014 study derived from six years of research by the 5 Gyres Institute estimated that 5.25 trillion plastic particles weighing some 269,000 tons are floating on the surface of the sea.^1^

At the same time, plastics in consumer products have become subject to increasing scrutiny regarding their potential effects on human health. Bisphenol A (BPA),^2^ a component of polycarbonate plastics and suspected endocrine disruptor, is one of the most widely known chemicals of interest. But BPA is only one of many monomers, plasticizers, flame retardants, antimicrobials, and other chemicals used in plastics manufacturing^3^ that are able to migrate into the environment.

**Figure:**
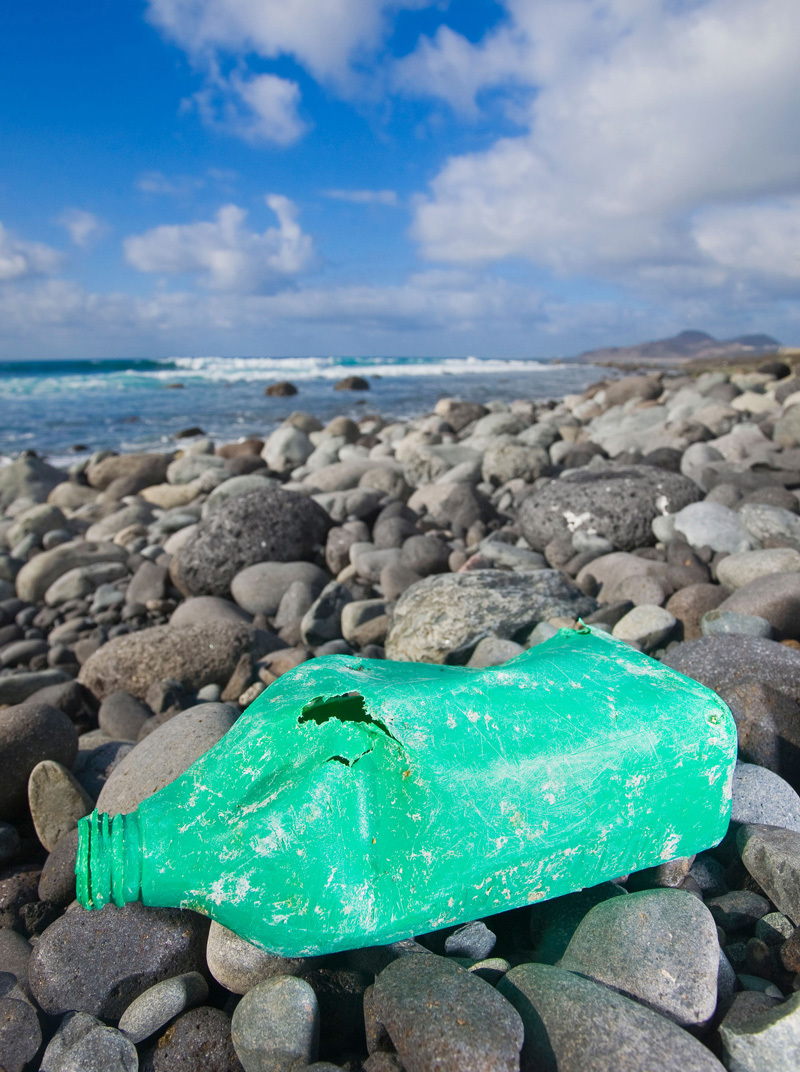
Investigators are researching whether consumption of plastic debris by marine organisms translates into toxic exposures for people who eat seafood. © Alex RM/Alamy

At the junction of these two lines of inquiry is an emerging third field that is in many ways even more complex and less well understood: investigating human exposures to and potential health effects of plastics that have entered the marine food chain. Studies have demonstrated plastics’ tendency to sorb (take up) persistent, bioaccumulative, and toxic substances, which are present in trace quantities in almost all water bodies.^4^ The constituents of plastics, as well as the chemicals and metals they sorb, can travel into the bodies of marine organisms upon consumption,^5^^,^^6^^,^^7^^,^^8^^,^^9^ where they may concentrate and climb the food chain, ultimately into humans. This topic has attracted interest and funding from the U.S. Environmental Protection Agency (EPA), the National Oceanic and Atmospheric Administration (NOAA), and the National Academy of Sciences (NAS), as well as researchers, nonprofit groups, and institutions around the world.

At this point “there are more questions than answers,” says Richard Thompson, a professor of marine science and engineering at England’s Plymouth University. Thompson coined the term “microplastics” in 2004^10^ and later undertook a three-year study of these particles in the marine environment for the UK’s Department of Environment, Food, and Rural Affairs.^11^^,^^12^^,^^13^ “From a human perspective,” he says, “at the moment I think there’s cause for concern rather than cause for alarm.”

Viewpoints on the human health risks of marine debris are nearly as complex as the underlying science, as was evident at an inaugural EPA and NAS symposium on the topic held in Washington, DC, in April 2014. In addition to myriad small details, the researchers in attendance considered an overarching question: Within the context of limited oceanographic research funding, the variety of other problems affecting ocean health (including overfishing and acidification), and the extent of humans’ daily and direct exposures to potentially harmful chemicals from consumer plastics and other sources—how concerned should we be about marine plastics as far as human health goes?

Researchers don’t yet have an answer, even if they believe they’re asking the right question. As EPA chemist Richard Engler concluded in a 2012 review, “While current research cannot quantify the amount, plastic in the ocean does appear to contribute to [persistent, bioaccumulative, and toxic substances] in the human diet.”^14^

## Plastic Vectors

The path from plastic pollution to chemical exposure through seafood is a long one, figuratively and often literally, and tracing all the individual steps in that theoretical journey is not the same as identifying human health effects, researchers say. Actual exposures, which are determined by innumerable variables along the way, including seafood consumption, still need to be quantified. Then these levels must be evaluated within broader contexts of consumer plastic use and environmental pollutant levels.

**Figure:**
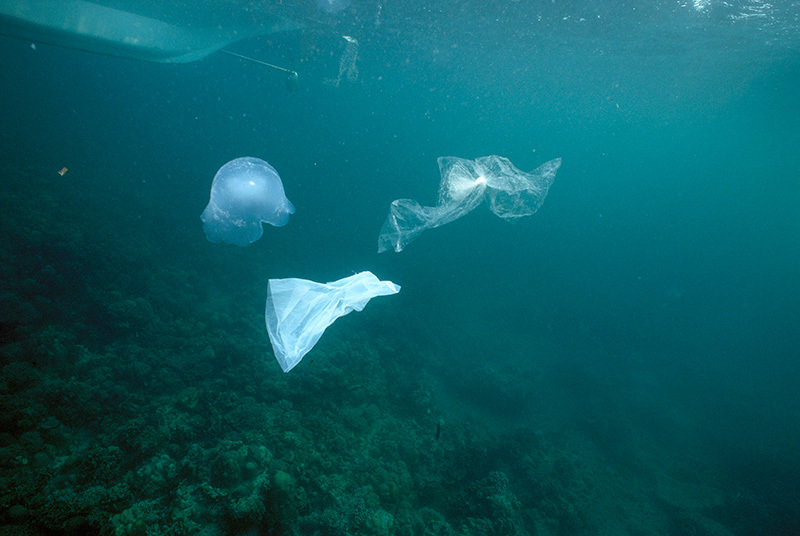
Different marine plastics resemble foods eaten at various trophic levels. These plastic bags look like the jellyfish eaten by turtles. © Norbert Wu/Minden Pictures/Corbis

**Figure:**
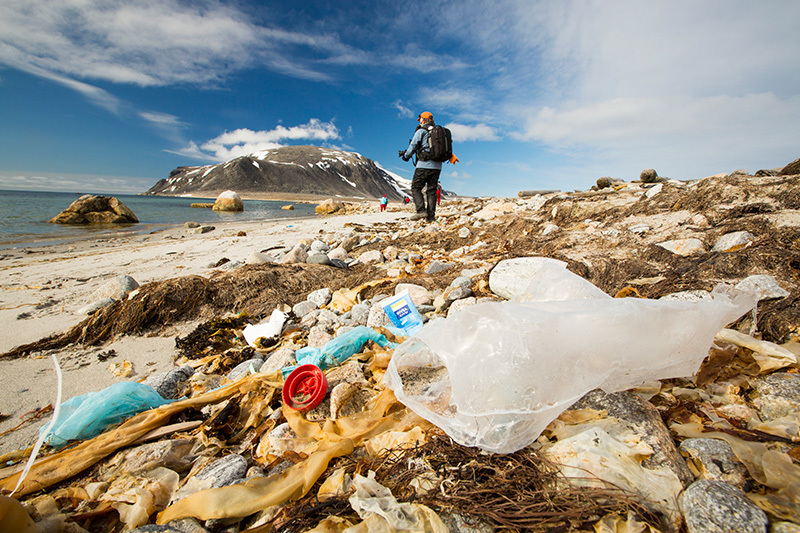
Plastic debris can travel far from its point of departure—this beach in Svalbard, Norway, for instance, is only about 600 miles from the North Pole. A 2014 study reported finding large quantities of microplastics frozen into Arctic ice.^52^ © Ashley Cooper/Corbis

Exposures to plastic debris have been clearly documented for marine organisms at all trophic levels (i.e., positions within the food chain), says Bradley Clarke, a lecturer at RMIT University in Melbourne, Australia. “What remains to be determined is whether this exposure increases the body burden of … marine organisms in the natural environment and if it does, by what magnitude,” Clarke says.

There is a lack of controlled experimental work completed on the topic, Clarke adds, and it’s very difficult to disentangle pollutant exposures and bioaccumulation via plastic versus food and environmental sources. Uncertainties also surround the transfer of plastic additives to marine organisms and resultant human exposures through seafood.

We do know that plastic has become nearly ubiquitous on the planet. It has washed up on the most remote beaches, amassed in distant gyres, and been discovered in the bodies of dead organisms from fish to birds to whales.^15^^,^^16^

Numerous efforts have sought to quantify the amount of plastics floating on or present throughout the ocean environment, and they’ve arrived at vastly different numbers. The 5 Gyres paper^1^ was preceded in July 2014 by a similar study suggesting that between 7,000 and 35,000 tons of plastic are floating on the ocean’s surface.^17^

Anna-Marie Cook, one of two EPA lead scientists investigating the potential health effects of marine plastics, believes that estimates calculated through the use of surface trawl nets, including both of the recent global studies, vastly underestimate the scope of the problem. “Slightly more than half of all plastic is negatively buoyant, meaning that it will sink upon reaching the ocean, either into the near-shore sediment environment or to the ocean floor,” she explains. “Surface trawls do not account for the fraction of plastic in sediments, on the ocean floor, or suspended past the top few feet of the water column.”

World plastics production has experienced almost constant growth for more than half a century, rising from approximately 1.9 tons in 1950^18^ to approximately 330 million tons in 2013.^19^ The World Bank estimates that 1.4 billion tons of trash are generated globally each year, 10% of it plastic.^20^ The International Maritime Organization has banned the dumping of plastic waste (and most other garbage) at sea.^21^ However, an unknown portion of the plastic produced each year escapes into the environment—instead of being landfilled, incinerated, or recycled^20^—and at least some of it eventually makes its way to sea.

Plastics that reach the ocean will gradually break down into ever-smaller pieces due to sunlight exposure, oxidation, and the physical action of waves, currents, and grazing by fish and birds.^22^ So-called microplastics—variably defined in the scientific literature and popular press as smaller than 1 or 5 mm in diameter—are understood to be the most abundant type of plastic in the ocean. The 5 Gyres authors found microplastics almost everywhere they sampled, from near-shore environments to the open ocean, in varying concentrations, and they estimated that particles 4.75 mm or smaller—about the size of a lentil—made up roughly 90% of the total plastic pieces they collected.^1^

**Figure:**
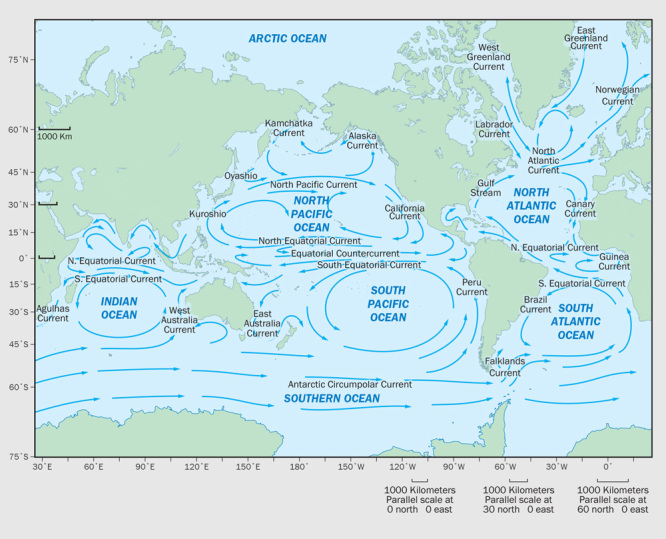
Ocean currents carry plastic debris into the five major ocean gyres. Thousands of tons of microplastics are estimated to bob in these gyres, but more than half of all plastic debris likely sinks upon reaching ocean waters. © Jane Whitney

But the degradation of larger pieces of plastic is not the only way microplastics end up in the ocean. Nurdles—the plastic pellets used as a feedstock for producing plastic goods—can spill from ships or land-based sources,^23^ and “microbeads” used as scrubbing agents in personal care products such as skin cleansers, toothpastes, and shampoos, can escape water-treatment facilities and pass into watersheds with treated water.^24^ (In June 2014, Illinois became the first U.S. state to ban the manufacture and sale of products containing microbeads,^25^ which have been documented in the Great Lakes^26^ and Chicago’s North Shore Channel.^27^)

Due to their hydrophobic nature, persistent organic chemicals—including polycyclic aromatic hydrocarbons (PAHs),^28^ polychlorinated biphenyls (PCBs),^29^ polybrominated diphenyl ethers (PBDEs),^30^ dioxins,^31^ and DDT^32^—have been shown to preferentially sorb to plastics when they encounter them in the ocean.^33^^,^^34^ Potentially thousands of such chemicals exist in the environment,^35^ but researchers are limited to screening for compounds they can actually identify, Bradley says.

The extent and rate of sorption can vary widely depending on the chemical, plastic type, and other variables, but plastic particles recovered from the ocean have been found to contain pollutant concentrations orders of magnitude higher than the water from which they were collected.^14^^,^^36^^,^^37^

Marine organisms throughout the food chain commonly consume plastics of various sizes.^38^^,^^39^ The tiniest microplastics are small enough to be mistaken for food by zooplankton,^40^ allowing them to enter the food chain at very low trophic levels. Some larger predators are thought to confuse nurdles (which typically measure less than 5 mm in diameter) with fish eggs or other food sources.^41^

Once plastics have been consumed, laboratory tests show that chemical additives and adsorbed pollutants and metals on their surface can desorb (leach out) and transfer into the guts and tissues of marine organisms.^14^ Some researchers speculate that chemicals already present in the organism may also be able to travel in the opposite direction by sorbing to plastics in the gut, depending on the concentration gradients. Yet neither process has been proven to occur in the natural environment.

**Figure:**
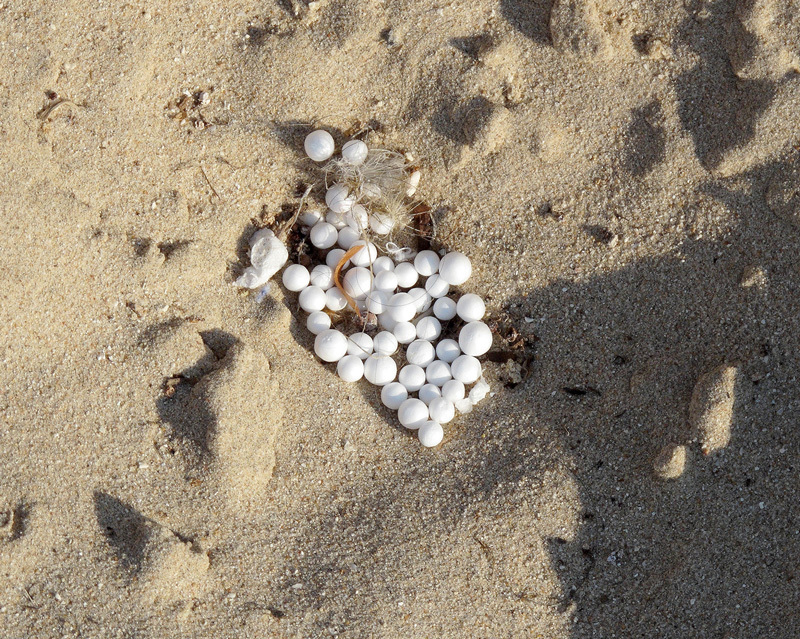
Small plastic pellets known as nurdles are used as a feedstock for producing plastic goods. In July 2012 Typhoon Vicente swept more than 165 tons of nurdles from a cargo ship off the coast of Hong Kong.^53^ © Nigel Cattlin/Science Source

**Figure:**
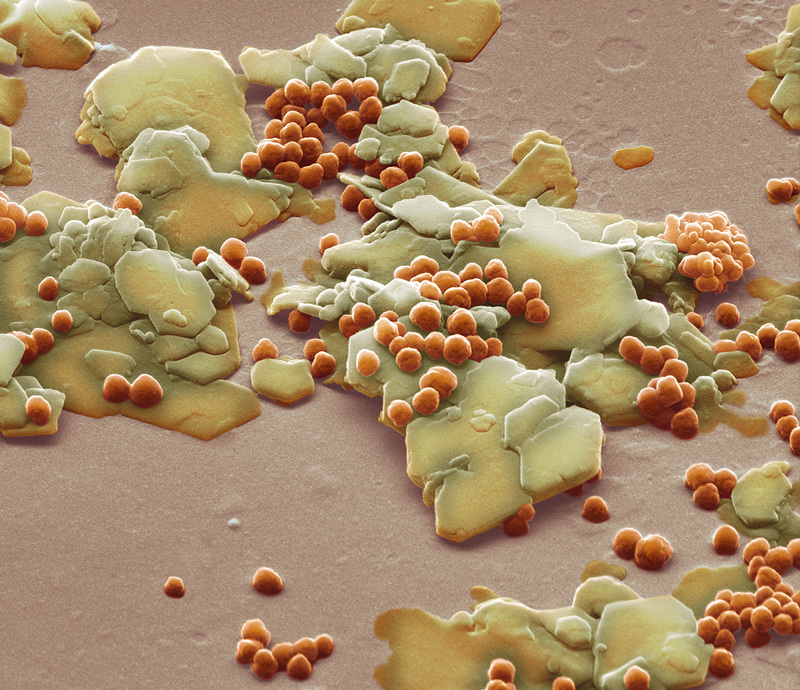
Polyethylene microbeads (orange, shown with yellow flakes of silica) are used as defoliants in many personal care products. In June 2014 Illinois became the first U.S. state to ban the manufacture and sale of products containing microbeads, which are small enough to slip through filters at wastewater treatment plants. © Steve Gschmeissner/Science Source

We already know that many chemicals of concern are present in the seafood we eat, particularly in higher-level predators such as tuna and swordfish.^42^ Research has shown that harmful and persistent substances can both bioaccumulate (or increase in concentration as exposures persist) and biomagnify (or increase in concentration at higher trophic levels) within organisms as they assume some of the chemical burden of their prey or environment. Yet again, no research has yet demonstrated the bioaccumulation of sorbed pollutants in the environment.

Three key questions remain to be determined. To what extent do plastics transfer pollutants and additives to organisms upon ingestion? What contribution are plastics making to the contaminant burden in organisms above and beyond their exposures through water, sediments, and food? And, finally, what proportion of humans’ exposure to plastic ingredients and environmental pollutants occurs through seafood? Researchers are moving carefully in the direction of answers to these questions.

## Human Health Questions

Among U.S. agencies, the EPA is delving into the science to answer key questions around marine plastics and human health. In addition to convening the April meeting and producing a forthcoming white paper on its findings, the agency collaborates with and directly funds researchers in the field. Staff from the EPA and the U.S. Fish and Wildlife Service are currently developing a risk assessment to quantify the chemical loading effects of plastic litter on marine life.^43^ And by 2016, the EPA plans to launch a similar long-term inquiry into effects on human health, including an evaluation of outcomes such as fetal formation, says Cook.

Any study of human health effects will likely depend on the cooperation of a subject community where many types of seafood are heavily consumed. “We have to have a potential threat and a potential receptor present in a location and a community who is willing to work with us on it,” Cook says. “There are a lot of repercussions to a community to find out that their food supply is potentially contaminated.” The agency also expects to award a new four-year marine debris research contract designed to gain a better understanding of the movement, distribution, and quantity of plastics off the remote northwestern Hawaiian islands.

**Figure:**
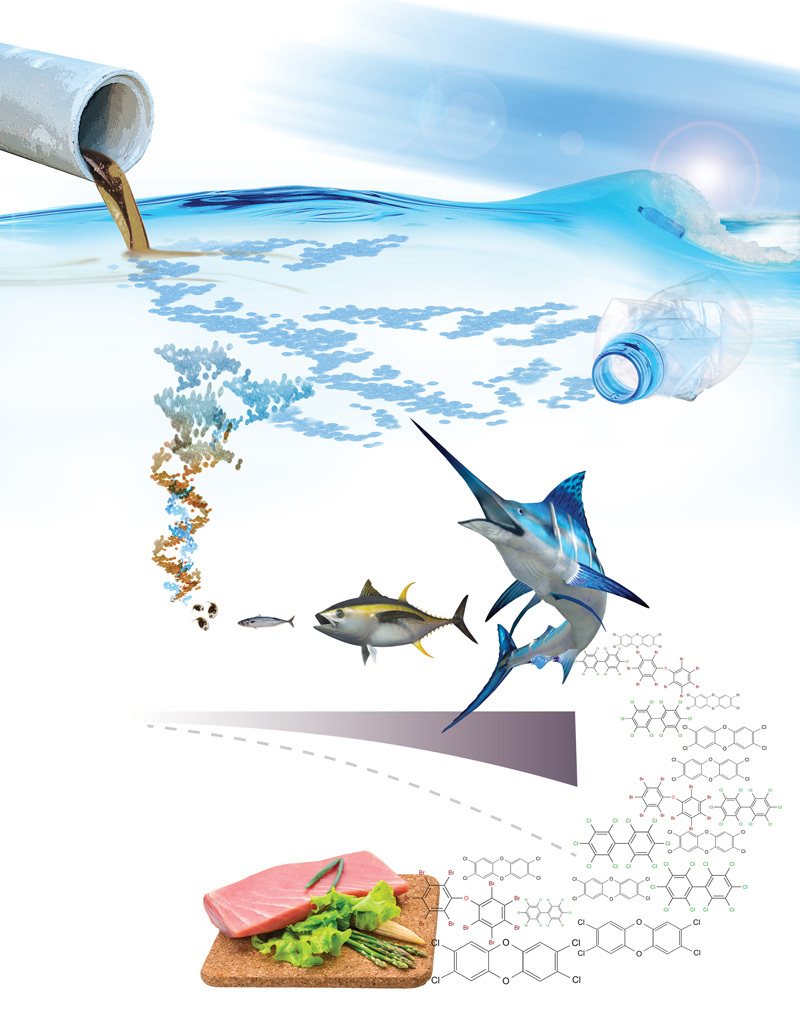
© Michael Northrop, Joseph Caspermeyer, and Rolf Halden/Biodesign Institute at Arizona State University

Researcher Chelsea Rochman of the University of California, Davis, collaborated with Cook and the EPA on a 2014 study that showed an association between concentrations of certain PBDEs in fish and levels of plastic debris accumulation in the South Atlantic Ocean.^44^ However, no such association was seen for concentrations of BPA, alkylphenols, alkylphenol ethoxylates, or PCBs in fish.^44^

Rochman is also working on a separate study funded through NOAA’s Marine Debris program. The aim of the NOAA study is to demonstrate for the first time the biomagnification in marine organisms of chemicals introduced via plastics. This highly controlled laboratory experiment involves feeding contaminated plastic pellets to mussels, feeding the mussels to sturgeon, and then testing levels of PCBs within the bodies of the sturgeon. Results are still awaiting analysis and publication.

One of Rochman’s collaborators on the project, researcher Mark Browne of the University of California, Santa Barbara, recently received a grant from the Australian Research Council for a three-year program addressing another question in the field: Beyond leaching chemicals, what do plastic particles do when they enter an organism? Browne showed in 2008 that microplastics sized 3.0 and 9.6 µm in diameter can travel beyond a mussel’s gut and into its circulatory system and hemocytes (immune cells), where they may remain for a relatively long period of time—in his study, more than 48 days.^45^ A 2012 study by another group showed that microplastics taken up by mussels resulted in a strong inflammatory response.^46^

The implications of these findings for humans that consume organisms containing microplastics are not yet understood. Browne says his team is currently working to develop a method to test human tissues for microplastics. “We think that’s going to be a big turning point,” he says.

**Figure:**
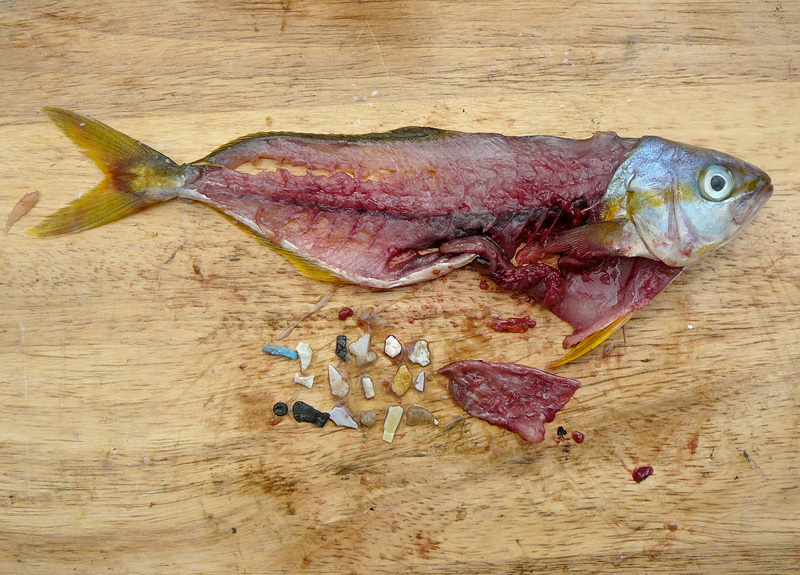
This rainbow runner had consumed 17 plastic fragments. Marine plastic pollution plays an unknown role in human exposures to toxic chemicals. Regardless of what that role may turn out to be, sources for this story believe we have options for realizing the benefits of plastics without the hazards of marine pollution. © 5 Gyres Institute

Ecotoxicologist Heather Leslie of VU University Amsterdam is among those concerned about the particle toxicity of microplastics themselves. Even without chemical hitchhikers, she says, plastic particles can induce immunotoxicological responses, alter gene expression, and cause cell death, among other adverse effects. “Exposed organisms then deal not only with chemical stress through multiple exposure routes, but also particle stress,” she explains. Leslie is currently studying the distribution and environmental fate of microplastics from cosmetics and other sources and potential toxicological effects on marine organisms in Europe’s multinational CleanSea Project.

A large body of literature about the mobility of nanoparticles offers a glimpse at how nano-size plastic particles may behave in the human body, Leslie says. “They can pass through the placenta and the blood–brain barrier and can be taken up in the gastrointestinal tract and lungs, potential sites where harm can occur,” she says. “There is a lot to learn about microplastics from the fields of particle toxicity and drug delivery technologies that apply to polymeric nanoparticles.”

In another example of ongoing research, Robert Hale, a professor at the Virginia Institute of Marine Science, has funding from both the EPA and NOAA to investigate how particle size, weathering, biofouling (the accumulation of living organisms on wet surfaces), and water characteristics including temperature, salinity, and organic carbon content influence both the sorption of organic contaminants to and the release of various additives from different types of microplastics.^47^ “You look at these simple parameters together, and it can get very complex,” Hale says. The EPA is particularly interested in evaluating the release of flame retardant additives from plastics, he notes, and may pursue development of a protocol to be used by manufacturers to provide data on chemical migration.

## A Matter of Perspective?

Government, academic, and independent sources interviewed for this article almost unanimously expressed a mix of skepticism and concern toward the thought of ocean plastics posing a human health risk. Without exception, they also advocated for further research. A common viewpoint is that although definitive evidence does not yet exist for real-world human health impacts tied to marine plastic debris, this doesn’t prove the hypothesis null, nor does it mean there aren’t other valid reasons to address the long-lived plastic litter that washes into the world’s oceans every year.

Many researchers pointed to the need to maintain perspective on the issue. Human exposure to microplastics and plastic additives is more likely to stem from intact goods prior to disposal than from seafood, Thompson says. Clothing fibers make up a large proportion of the microplastic found worldwide, says Browne,^48^ and even drinking water and foods such as honey can be contaminated with microplastics, according to Leslie.

Kara Lavender Law, a research professor of oceanography with the Sea Education Association in Woods Hole, Massachusetts, who collaborated with Richard Thompson on a recent summary of current knowledge about microplastics,^49^ says that while overfishing and direct exposure to consumer plastics concern her more than the marine-plastic pathway, the latter still warrants investigation. “I think it’s something worth working on,” she says. “Just because we don’t see it doesn’t mean it’s not there.”

In the case of plastic constituents thought to affect the human endocrine system, any level of exposure, no matter the route, may be potentially harmful, says Carol Kwiatkowski, executive director of The Endocrine Disruption Exchange. Endocrine disruptors have shown evidence of a nonlinear or nonmonotonic dose response,^50^ meaning tiny doses may have larger effects than mid-level doses.

“Anything that interferes with hormone action potentially has an effect at a very low dose, because the endocrine system is designed to function at very small doses,” Kwiatkowski says. “So it’s possible this pathway could bring some exposure. You’d have to find some evidence that the chemicals were being carried through marine organisms and making it into people.”

From there, she says, researchers would still need to learn how any such exposures relate to or interact with other exposures to endocrine disruptors, including rapidly metabolized chemicals such as BPA and phthalates, and longer-lived additives such as flame retardants. In other words, to what extent do all these exposures add up, and how does that cumulative exposure translate to health outcomes? “It’s difficult to study additive effects,” Kwiatkowski says. “But it’s very important research to conduct.”

Nonetheless, the end goal, sources say, is not to abandon the use of plastic. “The benefits of plastics can be realized without the need for emission [to the ocean],” Thompson says. “And for me that’s the tipping point for taking policy action.” New laws, for example, could require handling plastics more responsibly at the end of their useful life through recycling, proper disposal, and extended producer responsibility.

Rolf Halden, director of the Center for Environmental Security at the Biodesign Institute at Arizona State University, advocates for another solution: manufacturing more sustainable plastics from the start.^51^ “We need to design the next generation of plastics to make them more biodegradable so that they don’t have a long half-life, they don’t accumulate in the oceans, and they don’t have the opportunity to collect chemicals long-term,” he says. “There’s just no way we can shield people from all exposures that could occur. Let’s design safer chemicals and make the whole problem moot.”
