# Erratum: “New Link in the Food Chain? Marine Plastic Pollution and Seafood Safety”

**DOI:** 10.1289/EHP465

**Published:** 2016-07-01

**Authors:** Nate Seltenrich

Environ Health Perspect 123(2):A34–A41 (2015), http://dx.doi.org/10.1289/ehp.123-A34

In the following sentence, the amount of plastics produced in 1950 was incorrectly cited as “approximately 1.9 tons”:

“World plastics production has experienced almost constant growth for more than half a century, rising from approximately 1.9 tons in 1950…”

The correct number should have been 1.9 million tons.

*EHP* regrets the error.

